# Integrated cognitive behavioral treatment for substance use and depressive symptoms: a homeless case series and feasibility study

**DOI:** 10.1186/s40814-023-01305-2

**Published:** 2023-05-05

**Authors:** Olof Molander, Johan Bjureberg, Hanna Sahlin, Ulla Beijer, Clara Hellner, Brjánn Ljótsson

**Affiliations:** 1 PelarbackenErsta Diakoni, Social Welfare Office for the Homeless, City of Stockholm, Stockholm, Sweden; 2 Centre for Psychiatry Research, Department of Clinical Neuroscience, Karolinska Institutet, & Stockholm Health Care Services, Norra Stationsgatan 69, Plan 7, 113 64 Stockholm, Sweden; 3 Department of Clinical Neuroscience, Division of Psychology, Karolinska Institutet, Stockholm, Sweden

**Keywords:** Integrated cognitive behavioral treatment, Alcohol use disorder, Substance use disorder, Depressive symptoms, Homeless, Treatment first, Feasibility study

## Abstract

**Background:**

Homelessness is associated with high prevalence of psychiatric disorders such as substance use disorders, including alcohol use disorder, and depression.

**Methods:**

This case series and feasibility trial evaluated a novel integrated cognitive behavioral treatment (ICBT), which was adapted specifically for homeless individuals and developed to treat substance use and depressive symptoms simultaneously. The ICBT was delivered among four homeless individuals enrolled in the Treatment First program (a social services program where treatment is offered in conjunction with temporary transitional housing), who had access to stable and sober housing milieus.

**Results:**

The ICBT was rated high in expectancy of improvement, credibility, and satisfaction, with few treatment-related adverse events, and fairly high treatment retention. At 12 months follow-up, three of four participants were not homeless anymore. Some participants experienced short-term reductions in substance use and/or depressive symptoms.

**Conclusions:**

The study provided preliminary support that the ICBT can be a feasible and potentially effective treatment for homeless individuals with substance use and/or depressive symptoms. However, the delivery format within the Treatment First program was not feasible. The ICBT could be offered within the social services Housing First program instead (where permanent housing is offered before treatment), or to non-homeless individuals.

**Trial registration:**

The study was registered retrospectively at ClinicalTrials.gov (NCT05329181).

## Key messages regarding feasibility

- This case series and feasibility trial evaluated a novel integrated cognitive behavioral treatment (ICBT), which was adapted specifically for homeless individuals and developed to treat substance use and depressive symptoms simultaneously.
- The ICBT indicated good feasibility on most measures, i.e., expectancy of improvement, credibility and satisfaction, treatment retention, treatment-related adverse events, improved housing status, and potential reductions in substance use (including alcohol use) and symptoms of depression and anxiety.
- The delivery format within the social services Treatment First program was not optimal. Evaluation of the ICBT within the social services Housing First program, or to comorbid non-homeless participants with SUD and depression, might be considered.

## Background

More than 400,000 and 600,000 individuals are homeless in the European Union and the USA, respectively. Homelessness is seen as a complex interaction between individual and structural factors and is associated with poor mental and physical health [1–4] and increased mortality [5], as well as large costs for society. Definitions of homelessness vary across countries [4]. Sun et al. [6] proposed that the degree of homelessness could be measured and categorized into four groups with different living conditions: (1) Rough sleepers, i.e. homeless individuals who spend the night outdoors, for example in parks, under bridges, in cars or in stair-wells, (2) sheltered homeless, who spend the night in shelters, lodging houses, or so-called low threshold hostels where the guests are allowed to be intoxicated by some drug (including alcohol), (3) homeless in temporary residential institutions, including treatment institutions, where it is usually not allowed to be intoxicated, and (4) homeless close to housing, e.g., persons with a short-time sublease, or a long-term contract with a foundation for those with obstacles on the regular housing market, or someone living in a trial apartment, often arranged by social services, with an option of taking over the rental tenure. According to a similar definition as Sun et al. [6], 32,398 individuals in Sweden were homeless in 2011, and the number of individuals in long-term living arrangements offered by the social services had increased, which constituted almost half of the Swedish homeless population Board and of Health and Welfare [7].

The homeless population is a vulnerable group, specifically due to an increased risk of adverse health-related outcomes, including psychiatric disorders [4]. Swedish studies have estimated the prevalence of alcohol use disorder and/or substance use disorders (henceforth the term SUD will be used, also including alcohol use disorder) in the homeless population to 40–80%, with co-occurring psychiatric disorders between 42 and 50% [8], Board and of Health and Welfare [7]. Although psychiatric disorders have been proposed both as a potential cause and a consequence of homelessness [4, 6], little is known regarding the etiology of co-occurring depression and SUD in the homeless population [9]. In general, it is difficult to draw reliable etiological conclusions due to the chaotic and unstable environments that many homeless individuals are exposed to. Research on concurrent substance use and depressive symptoms among homeless populations is sparse, however, some previous findings have been reported. SUD have been found to be highly correlated with both initiation and persistence of homelessness [10]. Studies have shown that homeless individuals are specifically vulnerable to depression [9]. Social isolation, food deprivation, health- or relationship problems, SUD, low personal resources, self-esteem, or goal orientation are factors that have been discussed as possible explanations to the high prevalence of depression in homeless populations. Increased depressive symptoms, i.e., negative affect, have in general been associated with increased alcohol use [11], and with risk of dropout from SUD treatment among homeless individuals [12].

Swedish social services are typically operating according to the Treatment First program. In this working model, temporary transitional housing is offered using a continuous approach (e.g., non-drug-free emergency shelters or low threshold hostels, or sober residential institutions, trial or train apartments) alongside with psychiatric treatment, mainly for SUD. Access to permanent housing is conditional on “housing readiness” (e.g., being sober or drug-free) and adherence to treatment. Treatment First is the mainstream working model for homelessness in Sweden (i.e., treatment as usual) [10, 13, 14].

Standard mental health treatments are usually considered ineffective for homeless individuals with psychiatric comorbidity, due to insufficient outreach, lack of decent housing, and failure to address pending SUD [15]. Housing plays a key role for homeless individuals to respond to treatment. Access to stable, safe, and sober housing milieus is often seen as a prerequisite for starting treatment. Previous treatment studies for SUD have demonstrated effectiveness when homeless individuals have received simultaneous access to stable, safe, and sober housing milieus [16], and the treatments have been shown to be less effective when such housing milieus have not been offered [17]. According to international recommendations, treatment for homeless individuals should have an integrative approach for co-occurring mental illness and SUD, be tailored to the specific needs of the population, and coordinated in collaboration with other services, such as social workers [18]. According to our knowledge, no such cognitive behavioral treatment protocol exists today.

The aim of this study was to evaluate feasibility of a novel integrated cognitive behavioral treatment (ICBT), which was adapted specifically for homeless individuals and developed to treat substance use and depressive symptoms simultaneously. Feasibility was evaluated in a small sample of homeless individuals in the Treatment First program within the social services (where treatment is offered in conjunction with temporary transitional housing), who had access to “stable housing” (safe and sober housing milieus) during the treatment period. In this study, stable housing was operationalized as situations 3 and 4 according to the definition by [6], i.e., temporary residential institutions, e.g., treatment institutions where it is usually not allowed to be intoxicated, or close to housing, e.g., train or trial apartments arranged by the social services), because we did not want to exclude homeless participants in sober residential institutions. Treatment feasibility was investigated in terms of (1) expectancy of treatment improvement, credibility, and satisfaction,(2) number of completed treatment sessions and cancelled sessions; (3) occurrence of adverse events; (4) improved housing status; (5) reduction in substance use (including alcohol); (6) reduction in symptoms of depression and anxiety; and (7) treatment workload.

## Methods

### Design

A non-randomized ICBT feasibility study and case series of homeless individuals (*n* = 4), with measures administered pre and post treatment, weekly during treatment, and at 3-, 6-, and 12-month follow-up. Participants were recruited within a Treatment First program, using convenient sampling.

### Participants and study site

All study participants were clients at the social welfare office for the homeless, a specialized unit within the social services in Stockholm, Sweden. The participants were referred to outpatient ICBT at Pelarbacken, a specialized primary care center for homeless patients. Participants were included in the study if they (a) fulfilled the DSM-5 [19] criteria for AUD or SUD, (b) fulfilled the Swedish criteria for homelessness Board and of Health and Welfare [7] and had access to “steady housing” (defined as situation 3 or 4 according to [6], (c) were between 16–65 years old, (d) were able to read and write Swedish and were able to carry out treatment, 5–15 sessions together with homework assignments, and (e) had regular contact with a social worker at the social welfare office for the homeless. Exclusion criteria were (f) another primary psychiatric condition (e.g., bipolar disorder, psychosis, suicidal ideation), (g) failure to attend first two treatment sessions, and (h) other aggravating circumstances, for example violence in close relationships. Recruitment began in June 2016 and ended in January 2017. The last follow-up measure was administrated in July 2018.

In total, six homeless individuals were invited to participate in the study, of which five completed informed consent and were included. One participant started treatment but moved to another city. This participant was removed from the study, as the ethical permit did not cover other cities (or social services) than Stockholm. See Table 1 for baseline demographic characteristics of the four remaining participants.

**Table 1:** Baseline demographic properties of participants

Alias	Adam	Jennifer	Baako	Annelie
Age	≈60	≈50	≈50	≈30
Gender	Male	Female	Male	Female
Civil status	Single	Married	Single	Single
Own children (in contact)	Yes (no)	Yes (yes)	No	No
Number of children (ages)	2 (26, 23)	2 (39, 33)		
Employment (maintenance support)	No (yes)	No (yes)	Yes (no)	No (yes)
On sick-leave	Yes	Yes	No	No
Length of homelessness	11 years	2 years	7 years	8 years
Current housing status	Trial apartment^a^	Train apartment^a^	Trial apartment^a^	Trial apartment^a^
Presented complaints	Depression, anxiety, alcohol problems	Anxiety	Depression, isolation, social anxiety, gambling	Depression, social anxiety, anxiety, trauma, irritation, cannabis use
DSM-5 diagnoses^b^	APD, AUD^c^, GAD, MDD, PTSD	AUD^c^, PD	AG, APD, AUD^c^, MDD, PTSD, SAD, SUD^cd^	GAD, MDD, OCD, PDA, PTSD, SAD, SUD^cd^

*APD* antisocial personality disorder, *AG* agoraphobia, *AUD* alcohol use disorder, *GAD* generalized anxiety disorder, *MDD* major depressive disorder, *OCD* obsessive compulsive disorder, *PDA* panic disorder with agoraphobia, *PTSD* post-traumatic stress disorder, *SAD* social anxiety disorder, *SUD* substance use disorder

^a^Train and trial apartment, homeless housing situation 3 according to the definition bySun et al. [6]

^b^Assessed with the Mini International Neuropsychiatric Interview 7.0 [20]

^c^In a controlled milieu

^d^Cannabis use

### Measures

#### Acceptability

Perceived credibility and satisfaction of treatment were measured with the Credibility/Expectancy Questionnaire (CEQ; [21] and the Client Satisfaction Questionnaire (CSQ-8, [22], respectively. Higher scores indicate higher treatment credibility and satisfaction. The participants also reported adverse events using a self-report measure adapted for psychological treatment [23]. For each adverse event reported, participant also rated the discomfort caused by the event when it occurred, as well as residual discomfort (level of discomfort at the time of assessment). Ratings were made between 0 (“did not affect me at all”) and 3 (“affected me very negatively”).

The CEQ was administered after treatment session 2, and the CSQ-8 after treatment. The adverse event measure was administered after treatment, and at 3, 6, and 12 months following treatment cessation.

#### Housing status

Demographic questions were administered pre and post treatment, as well as during follow-up. The degree of homelessness was assessed with the questions “When was the last time that you had a housing of your own?” and”Where did you sleep last night?,” with response alternatives based upon the Swedish national definition of homelessness: “Outside”; “At a shelter”; “In a temporary (sober) residential institution,” “In a reference-based training or trial apartment,” or “In my own apartment (own lease)”. In addition, information of the participants’ housing status was collected from the registers of the social welfare office for the homeless at baseline and follow-up.

#### Substance use and psychiatric symptoms

The TimeLine Follow Back (TLFB; [24], a retrospective calendar instrument to assess days and quantity of alcohol and drug use, was used as primary measure for substance use. The TLFB have been found to have good psychometric properties in a homeless population [25]. In this study, alcohol and substance use was assessed using a retrospective 90-day calendar interview at baseline, and a retrospective 7-day measure was assessed weekly during treatment sessions. Number of units (alcohol or drug use per week) was reported as means per week during baseline and treatment. The TLFB was not administered at follow-up. The Patient Health Questionnaire (PHQ-9 [26], was used as primary measure for depressive symptoms. The following cut-off categories have been provided for the PHQ-9: none–minimal depression (0–4), mild depression (5–9), moderate depression (10–14), moderately severe depression (15–19), severe depression (20–27).

The Generalized Anxiety Questionnaire (GAD-7; [27], the Alcohol Use Disorders Identification Test (AUDIT, [28], and the Drug Use Disorders Identification Test (DUDIT [29], were used as secondary measures for anxiety, alcohol use, and drug use, respectively.

Primary and secondary measures were administered before and after treatment, as well as at 3, 6, and 12 months following treatment cessation. In addition, the TLFB and the PHQ-9 (1 week interval) were administered weekly during treatment.

### Procedure

Prior to inclusion, participants signed an informed consent, including consent for collaboration with the social welfare office for the homeless, and were assessed for psychiatric comorbidity with the Mini International Neuropsychiatric Interview 7.0 (MINI-7; [20]. The individual treatment sessions lasted between 30 and 60 min and were conducted at a location preferred by the participants. Two participants choose to receive the treatment at Pelarbacken, and two participants at another health care clinic and in their homes. In parallel to the treatment, participants received regular health care and social services interventions, such as housing supporters. The first author, a clinical psychologist, assessed and delivered the treatment as face-to face sessions for all participants except one. This participant was assessed with MINI-7 by a psychiatrist at Pelarbacken and had the first 9 treatment sessions delivered by a nurse at Pelarbacken who was trained by the first author, and the last 6 sessions delivered by the first author.

### Ethical considerations and safety procedures

The study was conducted in accordance with the Declaration of Helsinki Ethical Principles for Medical Research Involving Human Subjects and was approved by the Regional Ethics Board of Stockholm, Sweden (ref. no. 2015/2355–31/5). The following steps were taken to ensure participants safety and minimize dropout: (1) The ICBT was delivered at Pelarbacken, a specialized primary care center for homeless patients, with access to a team of health care providers such, physicians, nurses, and psychiatrists; (2) participants had an option to complete the ICBT sessions outside of Pelarbacken, for example as home visits, to reduce contact with alcohol and drug intensive milieus; (3) each ICBT session started with an “emergency list” targeting possible short-term issues that needed to be resolved to continue treatment for example lapses/relapses, medication, economical/housing complications; (4) participants had an option to have between session contact via phone or text messages, as well as weekly text message reminders of sessions; (5) participants that did not improve were offered referral to other treatment, e.g., specialized psychiatric treatment for anxiety; (6) participant’ names, stories and age were modified to ensure confidentiality, and lastly (7) the ICBT was conducted in collaboration with the participants social worker at the social welfare office for the homeless, optional parallel meetings were scheduled together with the participant and their social secretaires to ensure treatment confidentiality and secrecy. This last safety procedure was especially important as homeless individuals within the Treatment First model might risking losing their housing milieu, due to reported substance use.

### Analysis and missing data

Six-month follow-up assessment was missing for participant Baako, who was abroad at the occasion for measurement. Twelve-month follow-up was missing for Annelie, due to a relapse of drug use. In addition, assessment of GAD-7 was missing for Annelie at 6-month follow-up, due to a measurement error. These data were presented as not assessed. Three CSQ-8 items were missing for Annelie and were replaced with the respective mean CSQ-8 item score of the other participants. Alcohol, substance use, and depressive symptoms were assessed weekly during treatment sessions. Not assessed weeks were reported and replaced with last observation carried forward (see Fig. 3). As this was a non-randomized feasibility study with only four participants, outcomes were presented using descriptive statistics. TLFB units (alcohol or drug use) were presented in means per week, and self-report measures (PHQ-9, GAD-7, AUDIT and DUDIT) as total scores. All reported adverse events were reviewed and categorized as treatment, or non-treatment related, by author OM. Quantitative analyses were done using R Studio version 1.1.456 [30].

### Development of the intervention

The ICBT was developed as part of a collaborate treatment program between the social welfare office for the homeless and Pelarbacken. Initially during the program, homeless individuals were offered relapse prevention for substance use in a group format. Relapse prevention is a cognitive behavioral approach that aims to teach a variety of specific coping skills and decrease individual high-risk situations associated with relapse for substance use (e.g., [31]. However, relapse prevention was not considered optimal as the participants stated that they used alcohol and drugs in “all situations,” as a natural part of the homeless life. Furthermore, post treatment interviews and individual behavioral analyses of prior patients showed that the far most common reason for alcohol or drug use was coping with negative affect (46% reported this reason).

**Fig. 1:**
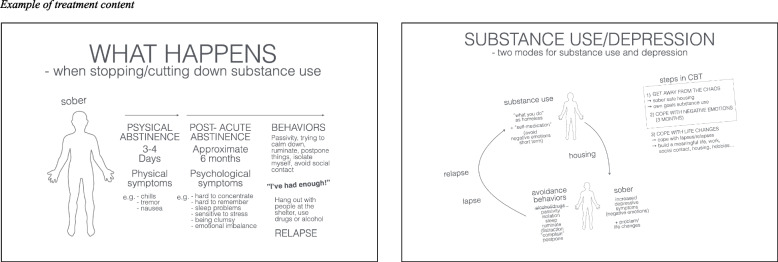
Example of treatment content

**Fig. 2:**
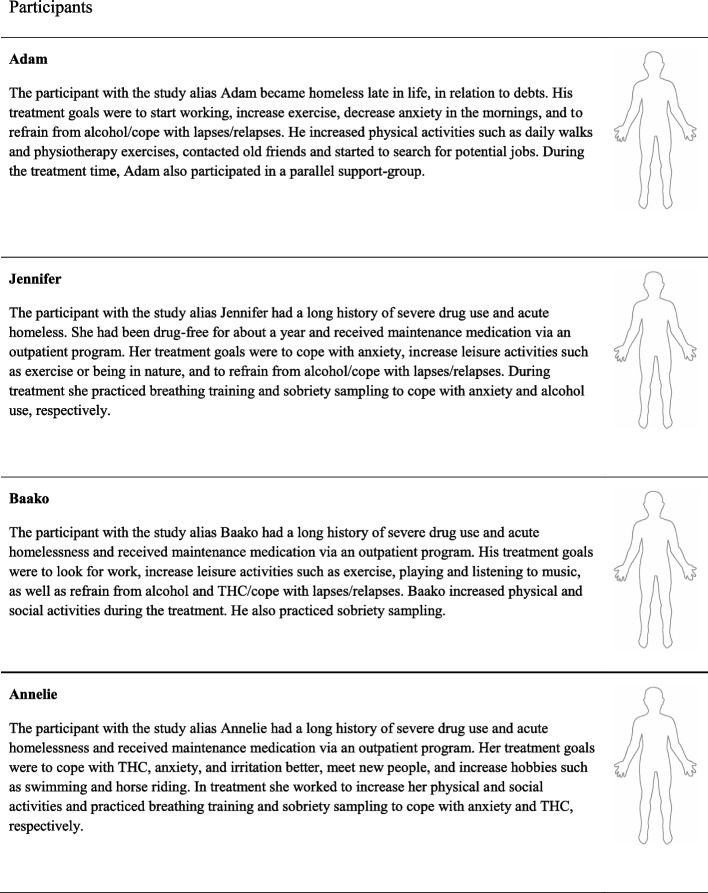
Participants

A novel ICBT was developed emanating from the following analysis (1) being homeless often implicates having lost contact with several important life areas, substance use might be the only reinforcing activity left; (2) common reactions are stress and depressive symptoms, and avoidance-based strategies such as passivity, isolation, avoidance of social contact, or substance use; (3) when decreasing substance use, a transient approximate 3-month period of increased “depression-like” symptoms occurs, which might lead to lapses or relapses (this period is also called post-acute abstinence, or protracted abstinence (e.g., [32])). The ICBT (5–15 sessions) was developed to extend over this time period, with the overall aim of participants to (1) access a stable, sober housing milieu, and decrease substance use; (2) learn strategies to cope with negative affect; and (3) learn strategies to cope with life changes, increase activities such as work, social contact, exercise, or leisure activities. Based on the post treatment interviews and individual behavioral analyses of prior patients, and previous research (e.g., [9, 11], we assumed that the intended mechanism of change (for both substance use and depressive symptoms) was reduction of avoidance-based behaviors in relation to negative affect/depressive symptoms. The ICBT included interventions from behavioral activation and relapse prevention (e.g., [31, 33], but did not involve any techniques derived from motivational interviewing (e.g., [34] (see Table 2 for treatment components, Fig. 1 for an example of the treatment content, and Fig. 2 for participants).

**Table 2:** Components of integrated treatment

Treatment interventions
Goal setting
Happiness scale, behavior analysis
Psychoeducation
Information regarding: (1) Homeless individuals health and life situation, (2) common reactions when decreasing substance use among homeless individuals, difference between lapses and relapses and how to cope with them, (3) common reactions to depressive symptoms among homeless individuals, (4) “self-medication”
Behavior activation^a^
Activity monitoring (activity diary), activity scheduling
Sobriety sampling
Strategies to postpone and cope with substance use
Breathing training
Strategies to cope with anxiety
Rumination
Strategies to identify and cope with rumination and urges

^a^Core treatment components

## Results

### Acceptability, treatment sessions, and adverse events

Mean CEQ ratings of treatment credibility (M = 8.38, Sd = 0.89, range 1–9) and expectancy (M = 75%, Sd = 16.90, range 0–100%) after session 2 were high. Satisfaction with treatment was rated in the higher range of CSQ-8, with a mean of 29 (Sd = 3.83) out of 32. Participants received in total 42 treatment sessions (M = 10.5, range 5 to 15). In total, 16 scheduled sessions (28%) were cancelled by the participants (range 0 to 6). In total, five adverse events were reported, of which two were categorized as treatment related (see Table 3).

**Table 3:** Treatment sessions and adverse events

	Total	Adam	Jennifer	Baako	Annelie
Number of sessions					
Completed treatment sessions, *n* (%)	42 (72%)	15 (100%)	15 (71%)	7 (58%)	5 (50%)
Scheduled dropout sessions, *n* (%)	16 (28%)	0 (0%)	6 (29%)	5 (42%)	5 (50%)
Adverse events	5				
Treatment related	2	“Lapse” (2/0)	“Panic attacks” (3/1)	-	-
Non-treatment related	3	-	“Had to take lung test for heart failure (3/2)	-	“Robbed by two guys” (2/0)
			“Anesthetized in respiration at hospital” (3/3)		

Number within parenthesis represents the level of discomfort (0–3) when the event occurred, and residual discomfort (at the time of assessment). For example, Adam reported a lapse and rated the level of discomfort as 2 and the residual discomfort as 0, which was categorized as a treatment-related adverse event

### Housing status, substance use, and psychiatric symptoms

**Fig. 3:**
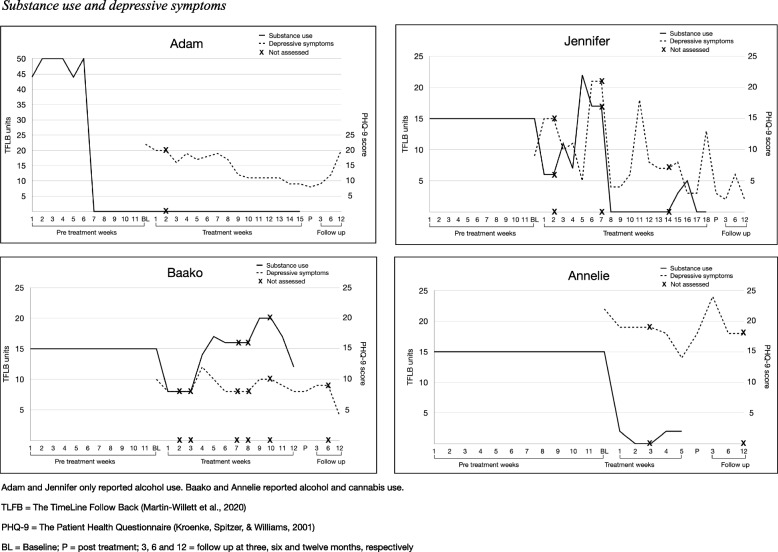
Substance use and depressive symptoms

No participant lost their housing during the study, due to, e.g., reported alcohol or drug use. Three participants had taken over the lease for their trial apartment and were thus not homeless anymore at 12 months follow-up. One participant, Jennifer, had moved from a training to a trial apartment. Overall, Annelie showed no improvements in psychiatric symptoms. She experienced a relapse for heavy drug use at the time for the 12 months follow-up and did not complete any of the measures, but if she had, all measures would probably have been elevated compared to baseline. All other participants seemed to show some reduction in anxiety from baseline to 12 months follow-up. Drug use patterns, assessed with the DUDIT, seemed similar at baseline compared to 12 months follow-up, or showed some reduction as with participant Baako. Depressive symptoms seemed to reduce continuously for Jennifer and Baako. Adam experienced a lapse or relapse of alcohol use at 12 months follow-up, as well as elevated depressive symptoms (see Table 4) (Fig. 3).

**Table 4:** Housing status and psychiatric symptoms

	Adam					Jennifer					Baako					Annelie				
	BL	P	FU3	FU6	FU12	BL	P	FU3	FU6	FU12	BL	P	FU3	FU6	FU12	BL	P	FU3	FU6	FU12
Housing status^a^	Trial (3)	Trial (3)	Trial (3)	Trial (3)	Own (4)	Train (3)	Train (3)	Train (3)	Train (3)	Trial (3)	Trial (3)	Trial (3)	Trial (3)	Trial (3)	Own (4)	Trial (3)	Trial (3)	Own (4)	Own (4)	Own (4)
Measure																				
PHQ9	22	8	9	12	20	9	3	2	6	2	10	8	9	NA	4	22	18	24	18	NA
GAD7	10	4	4	4	7	12	0	1	5	4	6	8	7	NA	2	19	21	21	NA	NA
AUDIT	0	0	0	0	15	5	7	0	0	1	10	20	14	NA	12	3	3	4	3	NA
DUDIT	0	0	0	0	0	0	0	0	0	0	8	8	6	NA	5	11	0	8	4	NA

^a^Register data from the Social Welfare Office for the Homeless, number within parentheses represents homeless housing situation according to the definition bySun et al. [6]

*BL* baseline, *P* post treatment, *FU3, FU6 and FU12* follow-up at 3, 6, and 12 months, respectively, *Train* training apartment, *Trial* trial apartment, *Own* own apartment (not homeless), *NA* measure not assessed, *PHQ9* The Patient Health Questionnaire [26], *GAD7* The Generalized Anxiety Disorder 7-item scale [27], *AUDIT* The Alcohol Use Disorders Identification Test [28], *DUDIT* The Drug Use Disorders Identification Test [29]

## Discussion

This study evaluated the feasibility of a novel integrated cognitive behavioral treatment (ICBT), adapted for homeless individuals, developed to treat substance use (including alcohol use) and depressive symptoms simultaneously. The ICBT was evaluated among four homeless individuals in the social services Treatment First program. In the Treatment First program homeless clients are offered a temporary accommodation, in conjunction with treatment. In the current study, all participants had access to stable, sober housing milieus, before starting the ICBT, which has been described as a condition for homeless individuals to be able to engage in treatment [35]. Previous substance use treatment studies among homeless individuals have shown good results when housing have been offered simultaneously [16], and been less effective when only treatment have been offered [17].

The ICBT was rated high in expectancy, credibility, and satisfaction, with few treatment-related adverse events. No participant dropped out of the treatment (not counting the participant who moved to another city and was removed from the study, due to geographical limitations in the ethical permit). In total, the number of completed treatment sessions was fairly high (*n* = 42, 72%), and the scheduled dropout sessions fairly low (*n* = 16, 28%). Previous studies have shown that treatment retention is difficult among homeless individuals, only about 25–33% complete treatments for substance use, even though the treatment programs are tailored especially to the needs of the population [36]. One possible explanation for treatment retention in this study could be that several recommended strategies to engage homeless individuals [37] were used in conjunction with treatment, such as offering a safe environment and outreach (home visits). Another possible explanation consists of the content of the treatment. Ibabe et al. [12] found that recent emotional stress among homeless individuals predicted less participation in substance use treatment compared to substance use alone, which predicted significantly more participation in treatment. In the current study, substance use and depressive symptoms were treated simultaneously using a behavioral activation approach (e.g., [33], thus addressing recent and emerging emotional stress, continuously. Finally, although this was a small, non-randomized feasibility study which did not permit causal inference, the results indicated that some participants experienced short-term reductions in substance use, and/or depressive symptoms. Overall, we conclude that the ICBT can be a feasible and potentially effective treatment for homeless individuals, which needs to be evaluated in larger controlled studies.

The delivery format within the Treatment First program was not optimal. Access to permanent housing within the Treatment First program is conditional on “housing readiness” and adherence to treatment (e.g., being sober or drug-free), while substance use might lead to losing a temporary housing milieu, such as trial or train apartments. Notably, three of four participants were not homeless at the end of the study, although the majority reported substance use during the ICBT, while they had access to sober trial and training apartments. As such, it is possible that the principle of “housing readiness” was applied less rigorously at the study site. Still, the condition of “housing readiness” within the Treatment First program can be seen as non-compatible with several treatment principles of the ICBT, such as voluntariness (own goals), or natural occurrences of lapses and relapses. In term of study workload, one clinical psychologist (OM) conducted practically all clinical assessments, treatment sessions, and scheduling, including a range of procedures designed to ensure treatment confidentiality and safety within the Treatment First program, such as home visits and cooperation with social workers and other health care clinics. These procedures might have increased participant safety and decreased dropout, cancelled visits and adverse events, but the workload surrounding treatment deliverance in this study seemed beyond regular clinical praxis. A further unexpected treatment obstacle was that half of the participants expressed that it was stressful to complete the ICBT, in relation to work, other health care visits, and mandatory measures within the Treatment First program, such as housing supporters. At the time of the study, the Housing First program was being implemented at the study site [14]. In the Housing First model, permanent housing is being offered as a first step [10, 13]. Continuous support is also offered, but housing is not contingent on adherence to psychiatric treatment or substance abstinence. Incorporating the ICBT into support systems of Housing First programs instead, for example in an Assertive Community Treatment team (an interdisciplinary Housing First team with healthcare professionals and social workers), could potentially increase feasibility from a treatment deliverance perspective. If so, it would be ensured that engagement in treatment is voluntary (i.e., not a conditioned measure to get access to housing). It would also be possible to coordinate and plan different interventions tailored to the participants needs and reduce the necessity of several safety procedures employed in this study, as well as reduce workload for the treatment deliverer. It would also enable possibility for “on demand” treatment interventions, to address, e.g., lapses and relapses, as support is continuously offered after participants get access to permanent housing. Unfortunately, Treatment First is still the mainstream working model [14], and only a monitory of the homeless population is being offered Housing First at the study site. Therefore, studies evaluating the ICBT within the Housing First program were not possible to conduct.

Although the focus of the ICBT was substance use and depressive symptoms, all participants in the current study also suffered from anxiety. This was not necessarily a problem, as behavioral activation (the main treatment component) may also be effective for anxiety disorders [33]. However, three of four participants suffered from post-traumatic stress disorder, for which this treatment was not sufficient. An alternative treatment approach to adapt for homeless individuals could have been prolonged exposure [38], which also have been evaluated in an integrated format for substance use [39].

This study was conducted among homeless participants receiving standard social service and health care interventions, and thus had high ecological validity. Several safety procedures were employed that ensured participant safety and treatment confidentiality within the Treatment First program, which was another strength of this study. Lastly, treatment was developed based upon behavior analysis of homeless participants and addressed an important gap identified in previous research [18]. Notably, comorbidity for SUD and depressive symptoms are not only common among homeless individuals. A recent meta-analysis among non-homeless community and clinical samples found that the pooled prevalence of any SUD in major depression was 25% (with AUD having the highest pooled prevalence of 21%) and that these comorbidity rates had not changed over the past three decades [40]. National treatment recommendations in Sweden [41] recommend integrated treatment for AUD and depression. No such cognitive behavioral treatment protocols exist today according to our knowledge. From this perspective, treatment development for specific vulnerable groups such as homeless individuals can be beneficial also for other patient populations.

Some study limitations are noted. Although the ICBT aimed to address substance use and depressive symptoms simultaneously, fulfilling MDD was not an inclusion criterion in the study, which would have been preferable. Another study limitation was that interviews were not conducted at follow-up due to practical reasons, and therefore the primary measure to assess substance use (TLFB) was not administered at these measure points. Further, depressive symptoms were not assessed at baseline using the same retrospective 12-week time period as the TLFB. Using a multiple baseline design [42] would have improved these measurement issues and ensured better experimental control. Finally, author OM developed the ICBT and administered the treatment and the data collection, thus allegiance bias cannot be ruled out.

Further studies could evaluate the ICBT in homeless samples in Housing First programs, or among comorbid non-homeless participants with SUD and depression.

## Data Availability

Dataset sharing is not possible due to integrity reasons, as the study participants were patients in routine health care.

## Abbreviations

APD: Antisocial personality disorder
AG: Agoraphobia
AUD: Alcohol use disorder
AUDIT: The Alcohol Use Disorders Identification Test
CEQ: The Credibility/Expectancy Questionnaire
CSQ-8: The Client Satisfaction Questionnaire
DUDIT: The Drug Use Disorders Identification Test
GAD: Generalized anxiety disorder
GAD-7: The Generalized Anxiety Questionnaire
ICBT: Integrated Cognitive Behavioral Treatment for substance use and depressive symptoms
MDD: Major depressive disorder
MINI-7: The Mini International Neuropsychiatric Interview 7.0
OCD: Obsessive compulsive disorder
PDA: Panic disorder with agoraphobia
PHQ-9: The Patient Health Questionnaire
PTSD: Post-traumatic stress disorder
SAD: Social anxiety disorder
SUD: Substance use disorders
TLFB: The TimeLine Follow Back
